# Supplementary material for: Legacy effects of historical grazing alter leaf stomatal characteristics in progeny plants
Source: PeerJ. 2020 Jun 17;8:e9266. doi: 10.7717/peerj.9266 (PMC7305771; doi:10.7717/peerj.9266)
Supplement: Supplemental Information 2 — (A) and (B), the adaxial leaf surfaces in enclosure and overgrazing Leymus chinensis respectively. (C) and (D), the abaxial leaf surfaces in enclosure and overgrazing Leymus chinensis, respectively. [file peerj-08-9266-s002.docx]

Supplementary Material


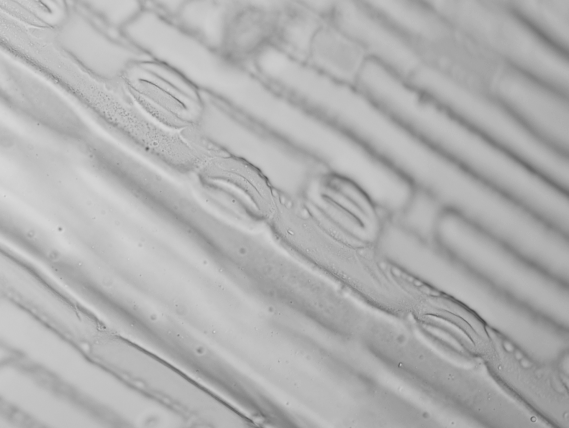

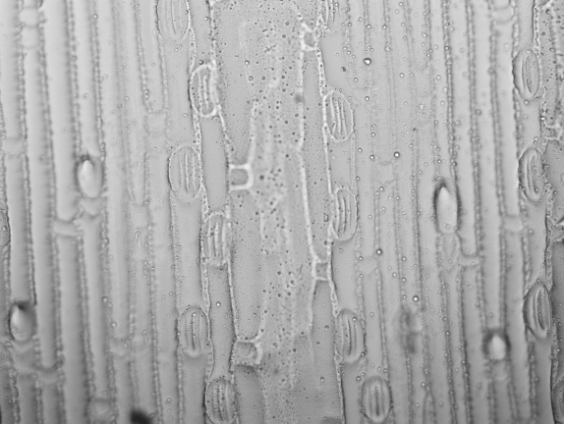


stoma

stoma

1. （B）


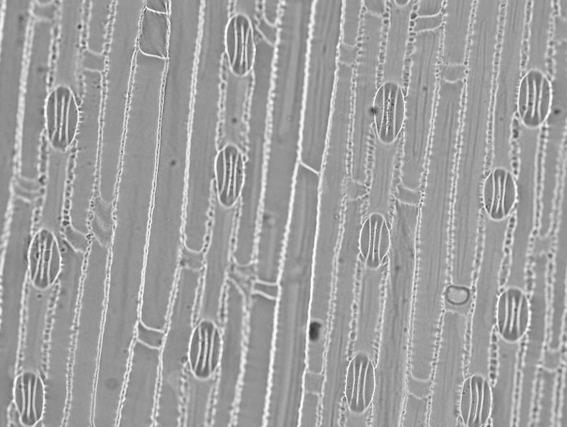

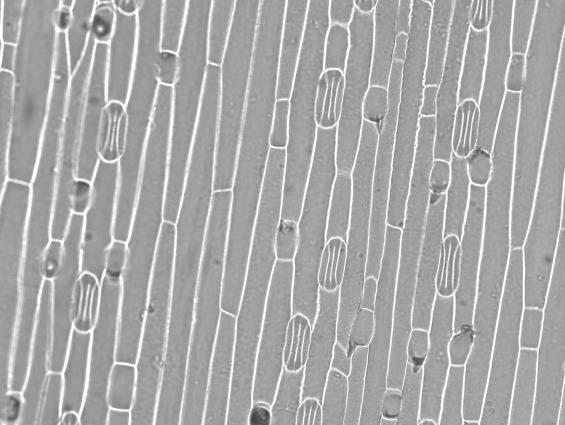


stoma

stoma

（C） （D）

Supplementary Figure S1: The stomatal characteristics (20×) in progeny plants. (A) and (B), the adaxial leaf surfaces in enclosure and overgrazing *Leymus chinensis* respectively*.* (C) and (D), the abaxial leaf surfaces in enclosure and overgrazing *Leymus chinensis* respectively*.*
