# Supplementary material for: Legacy effects of historical grazing alter leaf stomatal characteristics in progeny plants
Source: PeerJ. 2020 Jun 17;8:e9266. doi: 10.7717/peerj.9266 (PMC7305771; doi:10.7717/peerj.9266)
Supplement: Supplemental Information 3 [file peerj-08-9266-s003.docx]

Supplementary Material

**Supplementary Table S1:**

**qRT-PCR primer details of genes related to stomatal development and regulation in *Leymus chinensis* leaves**

| Gene ID | Primer No. | Primer Sequence（5'→3'） | Product size/bp |
| --- | --- | --- | --- |
| >c118857.graph_c0 | Lc-S2-f | TGCTTCTCACGGTTCCTTCG | 20 |
|  | Lc-S2-r | CAGTGGCAAGTCAACCTTAGTCC | 23 |
| >c92989.graph_c0 | Lc-S3-f | GTCCAAGGCGTGATGGGT | 18 |
|  | Lc-S3-r | TTGAACAGCGACAGGTAGTGC | 21 |
| >c128305.graph_c0 | Lc-S4-f | TACAGGGACGAACGCCACC | 19 |
|  | Lc-S4-r | CACAACACGGAGTAGGACGGA | 21 |
| >c93997.graph_c0 | Lc-S5-f | CTTCAGGCTTACTCGCTAT | 19 |
|  | Lc-S5-r | AGTTTTGTCTTCTTGGACG | 19 |
| >c113269.graph_c0 | Lc-S7-f | GATTGCTTGCATCATCTCGG | 20 |
|  | Lc-S7-r | GATAGGTGGCTGTGGTCGC | 19 |
| >c89156.graph_c0 | Lc-S8-f | GTCCATCCCCTCCGTTCC | 18 |
|  | Lc-S8-r | GCCCAATCCACCACCCTC | 18 |
| >c134962.graph_c0 | Lc-S9-f | CTCAACCCTGAAGAAGAAGGC | 21 |
|  | Lc-S9-r | TGTGATACTGTCAAACCCAACG | 22 |
| >c103838.graph_c0 | Lc-S10-f | TAGACATTCCAGATTCTCCAGCAC | 24 |
|  | Lc-S10-r | GCAGACCTCATTCCGTAAACAA | 22 |
| GenBank：HM623326.1 | Actin-f | ATTGTGCTCAGTGGTGGGTCA | 21 |
|  | Actin-r | CCAATCCAAACACTGTACTTCCTC | 24 |
